# An in silico MS/MS library for automatic annotation of novel FAHFA lipids

**DOI:** 10.1186/s13321-015-0104-4

**Published:** 2015-11-16

**Authors:** Yan Ma, Tobias Kind, Arpana Vaniya, Ingrid Gennity, Johannes F. Fahrmann, Oliver Fiehn

**Affiliations:** UC Davis Genome Center—Metabolomics, Davis, CA USA; Department of Biochemistry, Faculty of Sciences, King Abdulaziz University, Jeddah, Saudi Arabia

**Keywords:** In silico library, MS/MS, Lipids, FAHFA

## Abstract

**Background:**

A new lipid class named ‘fatty acid esters of hydroxyl fatty acids’ (FAHFA) was recently discovered in mammalian adipose tissue and in blood plasma and some FAHFAs were found to be associated with type 2 diabetes. To facilitate the automatic annotation of FAHFAs in biological specimens, a tandem mass spectra (MS/MS) library is needed. Due to the limitation of the commercial available standard compounds, we proposed building an in silico MS/MS library to extend the coverage of molecules.

**Results:**

We developed a computer-generated library with 3267 tandem mass spectra (MS/MS) for 1089 FAHFA species. FAHFA spectra were generated based on authentic standards with negative mode electrospray ionization and 10, 20, and 40 V collision induced dissociation at 4 spectra/s as used in in ultra-high performance liquid chromatography-QTOF mass spectrometry studies. However, positional information of the hydroxyl group is only obtained either at lower QTOF spectra acquisition rates of 1 spectrum/s or at the MS^3^ level in ion trap instruments. Therefore, an additional set of 4290 fragment-rich MS/MS spectra was created to enable distinguishing positional FAHFA isomers. The library was generated based on ion fragmentations and ion intensities of FAHFA external reference standards, developing a heuristic model for fragmentation rules and extending these rules to large swaths of computer-generated structures of FAHFAs with varying chain lengths, degrees of unsaturation and hydroxyl group positions. Subsequently, we validated the new in silico library by discovering several new FAHFA species in egg yolk, showing that this library enables high-throughput screening of FAHFA lipids in various biological matrices.

**Conclusions:**

The developed library and templates are freely available for commercial or noncommercial use at http://fiehnlab.ucdavis.edu/staff/yanma/fahfa-lipid-library. This in silico MS/MS library allows users to annotate FAHFAs from accurate mass tandem mass spectra in an easy and fast manner with NIST MS Search or PepSearch software. The developing template is provided for advanced users to modify the parameters and export customized libraries according to their instrument features.

**Electronic supplementary material:**

The online version of this article (doi:10.1186/s13321-015-0104-4) contains supplementary material, which is available to authorized users.

**Graphical abstract:**
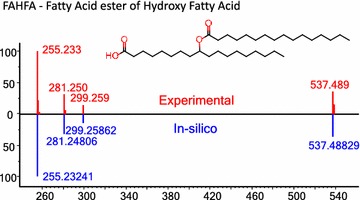
Example of experimental and in silico MS/MS spectra for FAHFA lipids

## Background

Recently, a novel lipid class named ‘fatty acid esters of hydroxyl fatty acids’ (FAHFA) was discovered in mice adipose tissues [1]. Specifically, a FAHFA comprised of palmitic acid (16:0) esterified to a 9-hydroxyl stearic acid (9-O-18:0), abbreviated as 9-PAHSA, was discussed as promoting anti-diabetic and anti-inflammatory effects [1]. PAHSA levels were shown to be highly correlated with insulin sensitivity in humans [1]. However, it remained unclear if other FAHFAs might exert similar effects, and how many different FAHFAs in total might be present in mammalian tissues or biofluids.

Currently only 21 external reference standards are commercially available. In order to enable extensive profiling and automatic annotation of FAHFA species, an MS/MS library with more structural diversity is needed. Today, such mass spectral libraries can be created by applying rules of fragmentation patterns on large in silico structure list, as we have previously shown for over 200,000 mass spectra in LipidBlast [2] for twenty-six common lipid classes such as (lyso) phosphatidylcholines, monogalactosyldiacylglycerols or triacylglycerols. LipidBlast itself has been applied for the annotation of lipids in mouse liver [3], rat urine/serum [4] and in various algae species [5, 6], demonstrating that this strategy enables rapid annotation of many molecular species from mass spectra [7]. LipidBlast templates use heuristic information of MS/MS fragmentation patterns to extend the range of in silico predicted mass spectra that can be used to discover species of novel lipid classes. An example has been shown for glucuronosyldiacylglycerol lipids in plants [8]. Here we used the modified LipidBlast templates to build an in silico MS/MS library for 1089 species of the novel FAHFA lipid class and demonstrate the applicability of this new library.

## Results and discussion

**Fig. 1:**
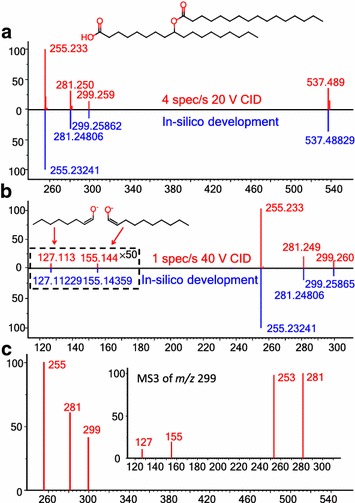
Experimental and in silico spectra of the model compound 9-PAHSA in negative ionization mode. **a** MS/MS spectra with collision energy of 20 V at 4 spectra/s acquired by QTOF; **b** MS/MS spectra with collision energy of 40 V at 1 spectrum/s acquired by QTOF, and **c** MS/MS and MS^3^ spectra acquired by LTQ with 20 % collision energy. The actual relative intensities for *m/z* 127 and 155 in figure **b**) are both 0.2 %

Negative mode electrospray in silico MS/MS spectra were modelled based on the reference spectra of 9-PAHSA under 10, 20, and 40 V collision induced dissociation (CID) voltages acquired with UHPLC-QTOF MS/MS profiling methods at 4 spectra/s (Fig. 1a; Additional file 1). Under these conditions, major FAHFA fragment ions include the precursor ion, the fatty acid fragment ion, and the hydroxyl fatty acid fragment ion including its dehydration product. No major differences in fragmentations were observed for either collision voltage, except for decreasing intensity of the precursor ion. Several isotopic peaks were observed for both precursor and fragment ions due to the insufficient isolation prior to the collision induced dissociation; however, such isotope ions were excluded from in silico fragmentation modeling since they could have been avoided with a narrower isolation width. Acquired 9-PAHSA spectra were compared to the published 9-PAHSA MS/MS spectrum and corresponding multiple reaction monitoring (MRM) fragmentation transitions [1]. While the major fragmentation ions observed in our laboratory were consistent with the published spectra, importantly, the positional fragments of the hydroxyl 18:0 fatty acid (*m/z* 127 and 155) were not observed. To generate those fragments (Fig. 1b), MS/MS experiments with a longer acquisition time of 1 spectrum/s were performed at 40 V CID, and *m/z* 127.133 and 155.144 were observed at relative abundance of 0.2 %. The intensity of such secondary fragmentation products could not be enhanced by increasing collision voltages on the MS/MS level with QTOF MS/MS acquisition time of 4 spectra/s. Indeed, these low abundant fragments were proven to be secondary fragmentation products of MS/MS ions by using MS^3^ fragmentation of the corresponding MS/MS ions, using direct infusion of the standards into a ThermoScientific Linear Ion Trap LTQ mass spectrometer (Fig. 1c).

To expand the overall structure space of the FAHFA structures that can be annotated by mass spectrometry, we used 33 fatty acids commonly found in mammalian cells, varying from 14:0–24:6 (Additional file 2) for both the free fatty acid and the hydroxyl fatty acid moieties [9–11]. 1089 general FAHFA structures were defined and 3267 in silico spectra were modelled based on the fragmentation pattern of 9-PAHSA observed at three collision energies. Since the positional information of double-bonds and hydroxyl groups could not be reflected by the reference spectra acquired with the fast 4 spectra/s QTOF MS/MS profiling method, such detailed information was not specified in the structures of FAHFAs. Therefore, the structures in this FAHFA profiling library are general, semi-characterized structures. To characterize the position of the hydroxyl group, we built a more specific in silico library based on the fragment-rich spectra at 40 V modeled from 1 spectra/s QTOF MS/MS acquisition time. According to a patent on FAHFA lipids [12], the hydroxyl group may be positioned on all carbons except for the terminal carbon. Correspondingly, 4290 structures with saturated hydroxyl fatty acids and saturated or unsaturated fatty acid esters were defined, and their in silico spectra were created based on the reference spectra of 9-PAHSA acquired with 1 spectrum/s MS/MS method. Due to a lack of published spectra or commercially available standards, modeling for unsaturated hydroxyl fatty acid residues was excluded. To further verify the position of the hydroxyl group we used relative retention time information. For the elution order of three commercially available OAHSA isomer reference standards, we observed increasing retention times when the hydroxyl group was positioned closer to the carboxylic acid moiety, with 12-OAHSA eluting at 6.07 min, 9-OAHSA at 6.21 min and 5-OAHSA eluting at 6.45 min under the conditions described in the experimental section. For building a reliable retention time database or even predicting retention times for other FAHFAs, a larger range of authentic reference standards would be needed.

**Fig. 2:**
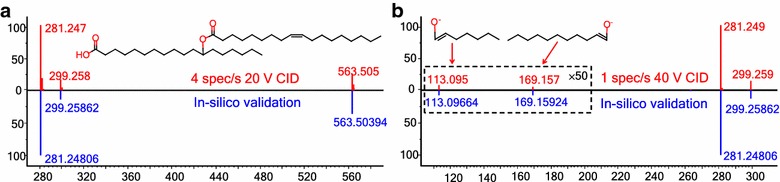
Experimental and in silico MS/MS spectra of one standard for validation 12-OAHSA in negative ionization mode. Spectra were acquired at **a** collision energy of 20 V at 4 spectra/s and **b** collision energy of 40 V at 1 spectrum/s. The actual relative intensities for m/z 113 and 169 are 0.2 and 0.1 %, respectively

The experimental MS/MS spectra of 5-OAHSA, 9-OAHSA and 12-OAHSA (Additional file 1) were used for validation of the in silico library as they had not been employed in the library generation. Using NIST MS Search, all general structures were correctly annotated by the in silico FAHFA library with a Reverse-Dot score of greater than 950. Figure 2 shows an example of the annotation of 12-OAHSA at 20 and 40 V using 4 and 1 spectra/s acquisition rates. For each case, the correct FAHFA was identified, and when using the longer MS/MS acquisition time, the correct isomer was identified, even when there were slight differences between predicted and experimentally observed ion intensities at specific collision induced voltages. As further validation, negative mode MS/MS spectra of eight published FAHFAs from the METLIN [13] database were also successfully annotated using the in silico library (Additional file 3).

**Fig. 3:**
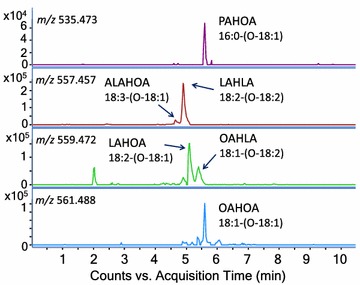
Extracted ion chromatograms (EICs) for FAHFAs found in negative ionization mode in egg yolk. The *m/z* tolerance was 0.005 Da

**Fig. 4:**
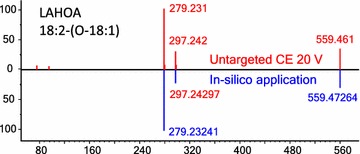
MS/MS spectra of FAHFA 18:2-(O-18:1) in negative ionization mode, annotated in the egg yolk by 4 spectra/s MS/MS method. Position of hydroxyl group is not specified. The experimental spectra have two extra peaks m/z 75.751 and 95.822, which may come from the background noise or other co-eluting compounds

To validate the usefulness of this new in silico FAHFA library, we analyzed complex lipids extracted from egg yolk and annotated FAHFAs by matching experimental to predicted MS/MS spectra. PAHSA isomer levels in egg yolk were measured using multiple reaction monitoring (MRM) in the previously published report [1], but no other FAHFA family members were reported in egg yolk. With the in silico library, we successfully annotated six abundant FAHFAs in egg yolk with <5 mDa errors for the precursor ions and Reverse-Dot scores greater than 900 for the MS/MS matching, including four FAHFA lipids that have never been detected before (Table 1; Fig. 3). As example for such novel FAHFA lipids, Fig. 4 compares the experimental to the in silico MS/MS spectra of FAHFA 18:2-(O-18:1), or LAHOA, at a scan rate of 4 spectra/s and 20 V collision energy. Here the positional fragments were not detected, suggesting that it is very challenging to obtain the positional information from complex mixtures in fast-scanning LC–MS/MS experiments. These examples demonstrate that in silico libraries such as the FAHFA library created here are suitable to annotate novel compounds detected in untargeted UHPLC-QTOF MS/MS profiling studies. We suggest that this library may be used in studies investigating the biological functions, regulation and distribution of FAHFAs.

**Table 1:** Information about FAHFAs found in egg yolk

Name	Abbrev.	RT (min)	*m/z*	Rev-dot
16:0-(O-18:1)	PAHOA	5.61	535.471	910
18:1-(O-18:1)	OAHOA	5.61	561.485	987
18:1-(O-18:2)	OAHLA	5.42	559.469	994
18:2-(O-18:1)	LAHOA	5.08	559.469	999
18:2-(O-18:2)	LAHLA	4.87	557.454	997
18:3-(O-18:1)	ALAHOA	4.67	557.454	957

*RT* retention time

*m/z* can be used for annotation in future LC-MS profiling experiments

## Conclusions

We developed an in silico MS/MS library for FAHFA lipids with a total of 7557 QTOF spectra in negative ionization mode. The new library enables users to automatically annotate FAHFAs in LC–MS/MS lipidomics profiling and can be therefore applied to further studies of this novel lipid class. The batch annotation process is very easy and fast using NIST MS PepSearch. We also provided the Excel template for users to adapt this library to their own instrument features and parameters and export customized libraries. The developed library and templates are freely available for commercial or noncommercial use under creative commons-by attribution (CC-BY) license and can be downloaded from http://fiehnlab.ucdavis.edu/staff/yanma/fahfa-lipid-library. The subset of 4290 spectra with defined structures (provided as InChI codes) is also available in Massbank of North America (MoNA) at http://mona.fiehnlab.ucdavis.edu/.

## Methods

### Experimental measurements of standards

FAHFA standards, including 9-PAHSA [16:0-(9-O-18:0)], 5-OAHSA [18:1-(5-O-18:0)], 9-OAHSA [18:1-(9-O-18:0)], and 12-OAHSA [18:1-(12-O-18:0)], were purchased from Cayman Chemical (Ann Arbor, MI). Stock solution for each lipid standard was prepared in ethanol at 1 mg/mL, and then diluted in methanol to 50 ppm for injection. LC–MSMS acquisition was performed by an Agilent 1290 HPLC coupled to an Agilent 6530 quadrupole time of flight (QTOF) mass spectrometer. A Waters Acquity CSH C18 column (2.1 × 100 mm, 1.7 μM) was used for separation. Mobile phase A consisted of 60:40 acetonitrile:water while mobile phase B consisted of 90:10 isopropanol:acetonitrile, both with 9.2 mM ammonium acetate. Column temperature was set to 65 °C and the flow rate was 0.6 mL/min. The following gradient was applied: 0–2 min from 15 % B–30 % B, 2–2.5 min from 30 % B–48 % B, 2.5–11 min from 48 % B–82 % B, 11–11.5 min from 82–99 % B, 11.5–12 min remain 99 % B, 12–12.1 from 99 % B–15 % B, 12.1–15 min re-equilibrate at 15 % B. 3 µL of each standard as well as a mixture of the four standards were injected. MS and MS/MS data was collected in negative ionization mode, in profile and centroid mode with a scan rate of 4 spectra per second. Multiple collision energies were applied, including 10, 20 and 40 V. In addition, MS/MS data was acquired specifically for *m/z* 537.489 and *m/z* 563.504 at acquisition time of 1 spectrum per second. MS/MS spectra were exported from Agilent MassHunter software to Mascot Generic Format (MGF) format. Additional confirmation was performed by MS/MS and MS^3^ experiments by direct infusion of 10 ppm standard solutions in methanol into a ThermoScientific Linear Ion Trap (LTQ) mass spectrometer at 5 kV electrospray voltage and 30 ms collision activation time with an activation Q set at 0.25, isolation width of 3 Da and the normalized collision energy for collision induced dissociation was set to 20 % for the acquisition of MS^3^ level spectra.

### In-silico development and validation

10, 20 and 40 V MS/MS spectra of 9-PAHSA were used for the development of the in silico library. Fragmentation patterns were manually investigated, and resulting *m/z* and abundance of all major peaks at each collision energy were included in the modified LipidBlast template. Molecular formula and accurate masses were calculated from chain lengths and degrees of unsaturation of the fatty acid and hydroxyl fatty acid residues, using the Exact Mass Calculator [14]. To expand the structure space of the library, a series of common fatty acids found in mammalian systems were added to the template, as fatty acid moieties [9–11]. Spectra information was calculated according to the model compound. Fatty acid structures were downloaded from the lipid metabolites and pathways strategy (Lipid MAPS) database [15]. Hydroxyl fatty acid and FAHFA structures were generated by ChemAxon Marvin 9.5.3 and JChem Reactor 9.5.3 [16]. VBA code of LipidBlast was modified to fit the new template and export the spectra to NIST MSP format. MSP file was then converted to NIST library by Lib2NIST [17] software and was ready to be used with NIST MS Search or NIST MS PepSearch software [18]. Other internal experimental MS/MS spectra as well as the external spectra from METLIN [13] online database were used for validation.

### Application: lipidomics of egg yolk

With slight modifications, lipid extraction of egg yolk was performed according to a previously published method [1]. Briefly, 300 µL egg yolk was added to 1200 µL citric acid buffer (100 mM sodium citrate, 1 M NaCl), followed by adding 1.5 mL of methanol and 3 mL of chloroform. The mixture was shaken by hand for 30 s, vortexed for 15 s, and centrifuged at 2200*g*, 4 °C for 6 min. The organic phase was dried under a gentle stream of nitrogen gas. The extracted lipids were reconstituted with 200 µL chloroform and loaded to a pre-conditioned SPE cartridge (500 mg silica, 6 mL, Thermo Scientific). Neutral lipids were eluted with 15 mL 5 % ethyl acetate in hexane followed by the elution of FAHFAs with 15 mL ethyl acetate. The FAHFA lipid fraction was dried under nitrogen gas and stored at −80 °C prior to LC/MS analysis. On the day of the experiment, the lipid extract was re-suspended with 30 µL of methanol and 5 µL was injected. The LC-MSMS method was similar to the method for the reference standard measurements, with a scan rate of 4 spectra per second and collision energy of 20 V. NIST MS PepSearch was used for the MS/MS search against the in silico FAHFA library.

## Additional files

10.1186/s13321-015-0104-4 Experimental MS/MS spectra of 9-PAHSA, 5-OAHSA, 9-OAHSA, and 12-OAHSA from Cayman Chemical, acquired with 10, 20, 40 V CID at 4 spectra/s and 40 V CID at 1 spectrum/s.
10.1186/s13321-015-0104-4 Common fatty acids included in the FAHFA in silico library.
10.1186/s13321-015-0104-4 Annotation results of the METLIN online reference spectra by the in silico library.
