# Supplementary figures and images for: An in silico MS/MS library for automatic annotation of novel FAHFA lipids
Source: J Cheminform. 2015 Nov 16;7:53. doi: 10.1186/s13321-015-0104-4 (PMC4646931; doi:10.1186/s13321-015-0104-4)

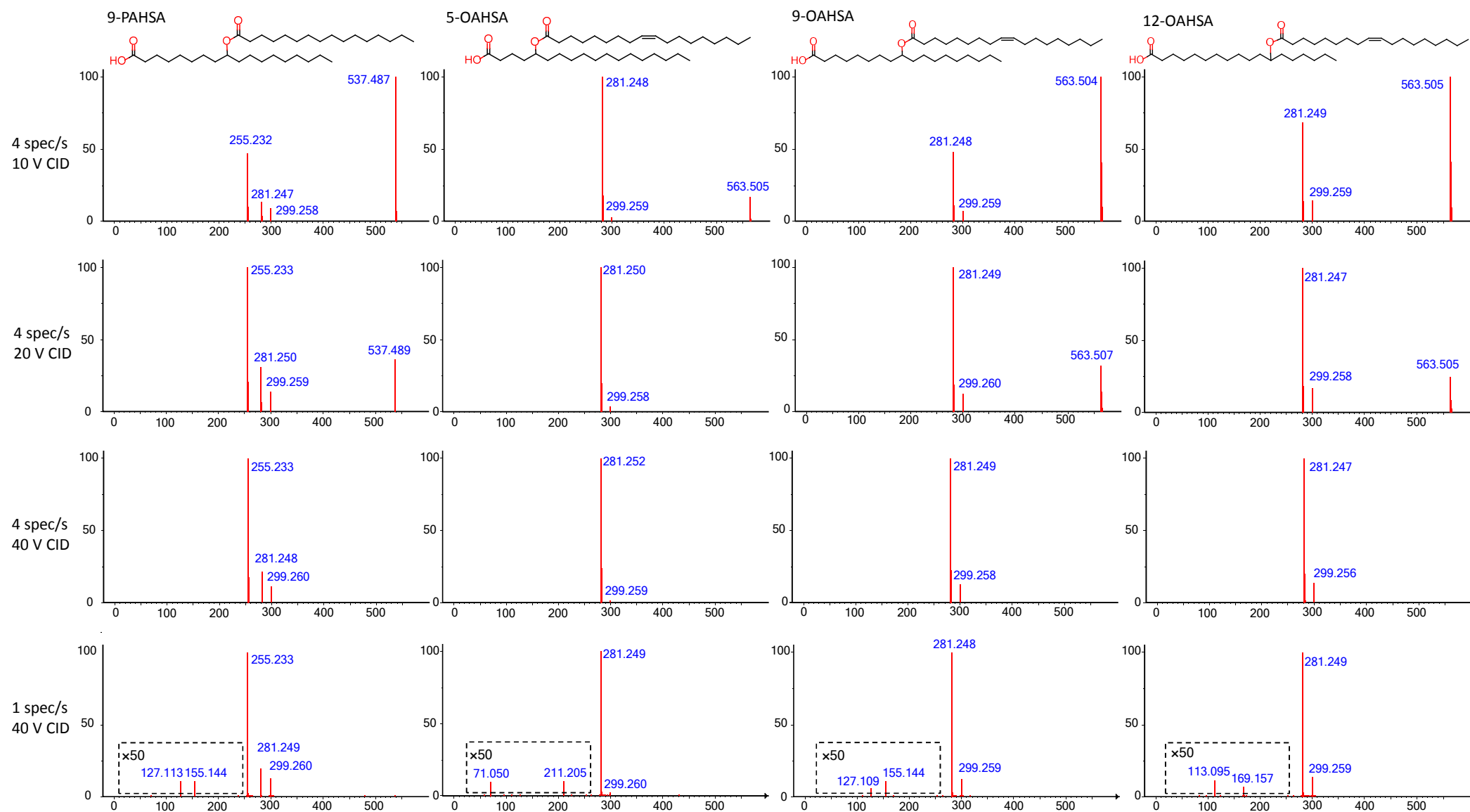

Supplement: Supplementary file 1 — 10.1186/s13321-015-0104-4 Experimental MS/MS spectra of 9-PAHSA, 5-OAHSA, 9-OAHSA, and 12-OAHSA from Cayman Chemical, acquired with 10, 20, 40 V CID at 4 spectra/s and 40 V CID at 1 spectrum/s. [file 13321_2015_104_MOESM1_ESM.pdf]
